# Effect of Antibiotic Eye Drops on the Nasal Microbiome in Healthy Subjects—A Pilot Study

**DOI:** 10.3390/antibiotics12030517

**Published:** 2023-03-04

**Authors:** Clemens Nadvornik, Martin Kallab, Nikolaus Hommer, Andreas Schlatter, Theresa Stengel, Gerhard Garhöfer, Markus Zeitlinger, Sabine Eberl, Ingeborg Klymiuk, Slave Trajanoski, Marion Nehr, Athanasios Makristathis, Doreen Schmidl, Alina Nussbaumer-Proell

**Affiliations:** 1 Department of Clinical Pharmacology, Medical University of Vienna, 1090 Vienna, Austria; 2 Division of Cell Biology, Histology and Embryology, Gottfried Schatz Research Center, Medical University of Graz, 8036 Graz, Austria; 3 Core Facility Computational Bioanalytics, Center for Medical Research, Medical University of Graz, 8036 Graz, Austria; 4 Department of Clinical Microbiology, Clinical Institute of Laboratory Medicine, Medical University of Vienna, 1090 Vienna, Austria

**Keywords:** antibiotic eye drops, gentamicin, ciprofloxacin, nasal microbiome, next-generation sequencing

## Abstract

Background: Antibiotic eye drops are frequently used in clinical practice. Due to the anatomical connection via the nasolacrimal duct, it seems possible that they have an influence on the nasal/pharyngeal microbiome. This was investigated by using two different commonly used antibiotic eye drops. Methods: 20 subjects were randomized to four groups of five subjects receiving eye drops containing gentamicin, ciprofloxacin, or, as controls, unpreserved povidone or benzalkonium chloride-preserved povidone. Nasal and pharyngeal swabs were performed before and after the instillation period. Swabs were analyzed by Illumina next-generation sequencing (NGS)-based 16S rRNA analysis. Bacterial culture was performed on solid media, and bacterial isolates were identified to the species level by MALDI-TOF MS. Species-dependent antimicrobial susceptibility testing was performed using single isolates and pools of isolates. Results: Bacterial richness in the nose increased numerically from 163 ± 30 to 243 ± 100 OTUs (gentamicin) and from 114 ± 17 to 144 ± 45 OTUs (ciprofloxacin). Phylogenetic diversity index (pd) of different bacterial strains in the nasal microbiome increased from 12.4 ± 1.0 to 16.9 ± 5.6 pd (gentamicin) and from 10.2 ± 1.4 to 11.8 ± 3.1 pd (ciprofloxacin). Unpreserved povidone eye drops resulted in minimal changes in bacterial counts. Preservative-containing povidone eye drops resulted in no change. A minor increase (1–2-fold) in the minimal inhibitory concentration (MIC) was observed in single streptococcal isolates. Conclusions: Antibiotic eye drops could affect the nasal microbiome. After an instillation period of seven days, an increase in the diversity and richness of bacterial strains in the nasal microbiome was observed.

## 1. Introduction

Bacteria, viruses, fungi, and other eukaryotic organisms are all part of the human microbiome, and as an ecosystem it is essential for maintaining homeostasis in tissues and organs including the skin, stomach, urogenital, oropharyngeal, and respiratory tracts [1,2,3,4,5,6].

Generally, the human or especially the nasopharyngeal microbiome received increasing attention over the last years, due to a variety of hypotheses linking dysbiosis to health and disease, as will be discussed in more detail in the following paragraphs. Furthermore, the nasopharyngeal area is colonized by commensal and pathogenic bacteria as well. Most dominating representatives of genera of the nasal microbiome of humans are *Staphylococcus*, *Streptococcus*, *Bifidobacterium*, *Corynebacterium*, *Dolosigranulum*, and *Moraxella*. Many pathogenic species, including, for example, *Streptococcus pneumonia*, *Haemophilus influenza*, and *Staphylococcus aureus*, also occur in healthy individuals, but mainly asymptomatically [7,8,9,10].

It has been found that the nasal microbiome is involved in several diseases of the upper respiratory tract, and it also seems to play a role in conditions of organs in anatomical proximity such as age-related macular degeneration or neurodegenerative diseases [11,12,13].

Studies have shown that patients with chronic rhinosinusitis with nasal polyps have significantly lower bacterial richness compared with patients without polyps [14,15]. Especially at a young age, dysbiosis of the microbiome may contribute to development of allergies, and patients with asthma have been found to have a different composition of the bacterial microbiome compared to healthy subjects [16,17]. Whether the existing imbalance of the microbiome is the consequence or the cause of diseases is still debatable. Nevertheless, the microbiome might play an important role in our health.

In clinical practice, ophthalmic topical antibiotics are often prescribed to treat bacterial infections and for prophylaxis after injuries and surgeries. Gentamicin and ciprofloxacin are the most commonly used substances [18]. Due to the anatomical proximity and direct connection via the nasolacrimal duct and the fact that up to 80% of ocular-applied medications diffuse into the systemic circulation via the highly vascularized nasopharyngeal mucosa, it seems possible that ophthalmic antibiotic therapy affects the nasopharyngeal microbiome. However, currently only few data on the effect of antibiotic eye drops on the nasal microbiome can be found in the literature [19].

In the present pilot study, we investigated the effect of one-week treatment with two different antibiotic eye drops on the nasal and the pharyngeal bacterial microbiome in healthy subjects. Even though the microbiome involves more species than only bacteria (e.g., fungi), we focused especially on the bacterial microbiome, as antibiotic eyedrops have been used in this study.

Here, the antibiotic formulations contained benzalkonium chloride (BAC) as a preservative (with potential additional antimicrobial effect). Thus, we decided to use two different topical lubricants as control, povidone eye drops with and without BAC, since also BAC could have an effect or possible influence on the microbiome as shown in other studies [20]. The purpose of this pilot study, therefore, was also to investigate whether BAC alone has an effect on the nasal or pharyngeal microbiome.

The primary objective of this work was to provide a first insight into this topic and gain preliminary results by conducting a pilot study, which should further serve as an outlook for subsequent studies including a higher number of healthy volunteers.

In this study, next-generation sequencing was applied. This method has the advantage to provide information on a broad variety of the bacterial microbiome deep down to the phylogenetic level, rather than merely on pre-selected species as is the case for culture-based technologies [21,22,23,24].

## 2. Materials and Methods

The study protocol was reviewed by the Ethics Committee of the Medical University of Vienna and approved by the relevant authorities. It was conducted in compliance with the guidelines of the International Council for Harmonization–Good Clinical Practice (ICH-GCP) and the Declaration of Helsinki. Written informed consent was obtained from all study participants prior to participation.

Subjects: Only healthy subjects with normal ophthalmic findings were included into the randomized, single-masked, controlled parallel group pilot study. The exact inclusion parameters for the study were: men and women aged between 18 and 45 years, normal results of the ophthalmic examination, and no use of topical eye or nasal drops in the last three months. All subjects had to pass a screening examination four weeks up to one day before the first study day. Screening included medical history, an ophthalmic examination, and a pregnancy test in women with childbearing potential.

Subjects were excluded if they fulfilled one or more of the following criteria: regular use of medication, abuse of alcoholic beverages or drugs, participation in a clinical trial in the three weeks preceding the study, treatment with any drug (except intake of hormonal contraceptives) in the previous three weeks, treatment with topical or systemic antibiotics within eight weeks before inclusion, symptoms of a clinically relevant illness in the three weeks before the first study day, history or presence of gastrointestinal, liver, or kidney disease or other conditions known to interfere with distribution, metabolism or excretion of the study drugs, known hypersensitivity to any of the components of the study drugs, pregnancy or breast-feeding, and no effective contraception in women of childbearing potential. Care was taken to ensure that no medications or vaccinations were taken by the subjects during study participation.

Study groups and Eye drops: The subjects were randomized to receive either Gentamicin (Gentax eye drops, Agepha Pharma, Senec, Slowakia), Ciprofloxacin (Ciloxan eye drops, ALCON Ophthalmika GmbH, Wien, Austria), Povidone-unpreserved (Protagent SDU eye drops, ALCON Ophthalmika GmbH, Wien, Austria), or Povidone-preserved (Oculotect fluid eye drops, Thea Pharma GmbH, Annonay, France) in a 1:1:1:1 ratio.

Every healthy subject in each group had to administer four drops daily for 7 ± 1 days with at least two hours between instillations.

First a pilot study with 20 subjects was performed, which is described within this manuscript. Based on the results of this pilot study, the control for the main part of the study will be chosen, depending on the effect on the bacterial microbiome. The results of the pilot study should provide useful data to adjust the sample size for the main study part.

Description of Study Protocol: For the included healthy subjects, two study days (Visit 2 and 3) were scheduled. On the first study day (Visit 2), the following measurements were performed: assessment of adverse events, pregnancy test in women with childbearing potential, nasal swabs, pharyngeal swabs, and an ophthalmic examination. On study day 2 (Visit 3), 7(±1) days later, all the measurements and assessments were performed as described for study day 1.

Sample collection techniques: Nasal and pharyngeal swabs: At each study visit, three nasal swabs were taken from the right nostril and three from the pharynx by using the “BD™ culture swab” (BD Diagnostics, Heidelberg, Germany), and samples that were used for microbiome analysis were stored at −80 °C until further processing. Samples for microbiological analysis and antibiotic susceptibility testing were immediately transferred to the microbiological laboratory and were processed promptly as described below.

Nasal and pharyngeal microbiome analysis: Samples stored at −80 °C were sent to the Core Facility Molecular Biology of the Medical University of Graz. There, further analysis took place according to published protocols [25]. Briefly, total DNA isolation was performed with the MagNA Pure LC DNA III Isolation Kit (Bacteria, Fungi) (Roche, Mannheim, Germany) in a MagNA Pure LC 2.0 instrument according to the manufacturer’s instructions. Then, 500 µL MagNA Pure Bacteria Lysis Buffer (Roche, Mannheim, Germany) was added to the samples and incubated according to the manufacturer’s instructions. For mechanical lysis, samples were bead beaten in MagNA Lyser Green Bead tubes (Roche, Mannheim, Germany) at 6500 rpm for 30 s two times in a MagNA Lyser instrument (Roche, Mannheim, Germany). Nucleic acids were eluted in 50 µL according to the manufacturer’s instructions, and samples were stored at −20 °C until PCR analysis [26,27,28].

16S targeted PCR amplification was performed in triplicate using FastStart High Fidelity PCR system (Roche, Mannheim, Germany), amplifying the hypervariable region V4 with the primers 515F (GTGYCAGCMGCCGCGGTAA) and 926R (CCGYCAATTYMTTTRAGTTT). PCR amplification was performed for 30 cycles according to published procedures (Klymiuk et al. 2022), and triplicates were pooled, indexed, and purified. Then, the library was sequenced on an Illumina MiSeq desktop sequencer (Illumina, Eindhoven, Netherlands) with v3 chemistry for 2× 300 cycles. FastQ files were used for data analysis.

Microbiological MALDI-TOF analysis and antibiotic susceptibility testing: Swabs were inoculated and incubated on blood agar plates at 37 degrees Celsius under aerobic conditions, and further bacterial strains were determined by MALDI-TOF. Matching isolates were grouped to pairs (before and after treatment) if present. Minimal Inhibitory Concentration (MIC) testing with broth-microdilution against gentamicin and ciprofloxacin was performed for each isolate of *Staphylococcus epidermidis* before and after treatment. Susceptibility testing of *Streptococcus* spp. for both above-mentioned antibiotics was performed by E-Tests for each paired isolate and in a pool (all isolates within one visit from each the nose and the pharynx) have been conducted. Additionally, to screen for other potential antibiotic resistances, antibiograms were evaluated. The obtained bacterial isolates were identified by microbiological analysis (MALDI-TOF), and antibiotic susceptibility testing was performed regarding the EUCAST guidelines (https://www.eucast.org/ast_of_bacteria/, accessed on 11 January 2022).

NGS data analysis: FastQ raw data from Illumina Sequencing were used for data analysis. Quantitative Insights into Microbial Ecology (QIIME2 Version 2018.4), a bioinformatic pipeline integrated in the open-source web-based platform Galaxy (https://galaxyproject.org/, accessed on 11 January 2022) hosted on the MedBioNode HPC cluster of the Medical University Graz, Austria (https://galaxy.medunigraz.at/, accessed on 11 January 2022), was used to analyze the final sequence files. Shortly, paired-end raw forward and reverse reads were quality-filtered, de-noised, de-replicated, merged, and checked for chimeras using DADA2 denoise pipeline with optimized parameters: p-trunc-len-f: 260, p-trunc-len-r: 220, p-trim-left-f: 19, p-trim-left-r: 20 and p-max-ee: 2.0. The outcome of DADA2 was further used for alpha and beta diversity analysis in QIIME2. Downstream statistical analysis was performed in R version 4.0.2 (R Core Team, 2020) accompanied with the packages vegan, ggplot2, and psych. Paired-end FASTQ raw sequence reads were uploaded to the NCBI sequence read archive (SRA) and can be accessed under the BioProject ID PRJNA851930.5.

## 3. Results

A total of 20 healthy subjects were included into the study. Mean age was 27 ± 6 years, 16 subjects were female and 4 were male. Subjects were evenly distributed between the study groups regarding age and sex (Table 1).

From the 4,443,738 raw reads, 3,486,684 reads were used for further analysis after quality filtering and trimming. For Alpha diversity calculations, two different parameters were obtained from the data, “bacterial richness” and the “phylogenetic diversity index” (pd) in the nasal and pharyngeal microbiome. To describe the bacterial richness, bacteria were categorized based on their sequence similarity, and the number of microorganisms in the microbiome were expressed in operational taxonomic units (OTUs). The phylogenetic diversity index is described by a quantitative measurement of the number of nodes in a phylogenetic tree that indicates how many different species are present in the data set.

**Figure 1:**
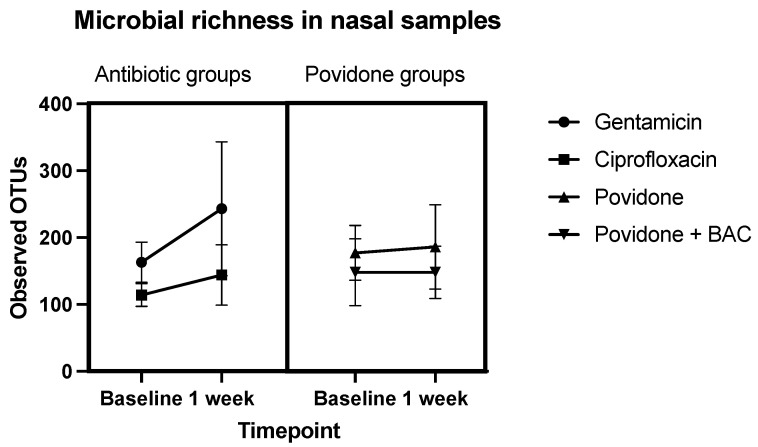
Microbial richness in nasal samples. Comparison of OTUs before and after treatment (Baseline: before instillation of eye drops, 1 week: after 7 days’ instillation). Sequences are clustered according to their similarity to another. Group 1 received gentamicin eye drops, group 2 received ciprofloxacin, group 3 received topical lubricants containing povidone only, group 4 received topical lubricants containing povidone and BAC.

Sequencing results provided information about the diversity and richness of the different sample sites. In the gentamicin group (group 1), bacterial richness in the nose increased numerically from 163 ± 30 to 243 ± 100 OTUs and in the ciprofloxacin group (group 2) from 114 ± 17 to 144 ± 45 OTUs. Unpreserved povidone eye drops resulted in minimal numerical changes in bacterial counts (from 177 ± 41 to 186 ± 63 OTUs). Preservative-containing povidone eye drops resulted in no change (148 ± 50 vs. 148 ± 39 OTUs). A schematic representation of these results is shown in Figure 1.

**Figure 2:**
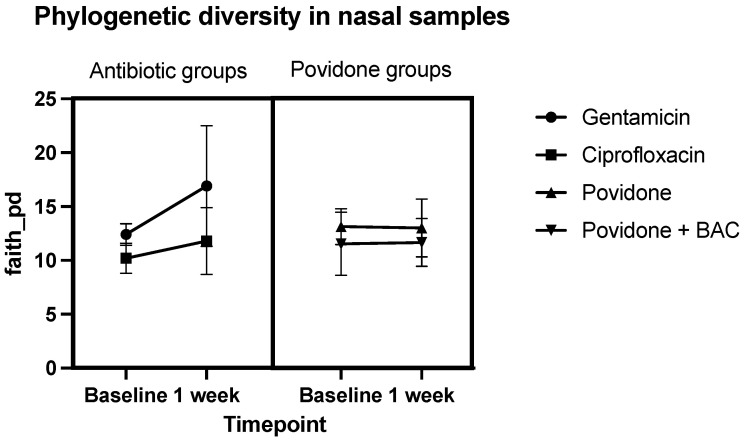
Faith_pd in nasal samples, meaning the sum of the branch lengths of a phylogenetic tree connecting all species in the target assemblage. Comparison of the phylogenetic diversity index before and after treatment (Baseline: before instillation of eye drops, 1 week: after 7 days’ instillation). Group 1 received gentamicin eye drops, group 2 received ciprofloxacin, group 3 received topical lubricants containing povidone only, group 4 received topical lubricants containing povidone and BAC.

In both antibiotic groups, the sequence analysis demonstrated that after 7 ± 1 days’ instillation, the phylogenetic diversity index (pd) of different bacterial strains in the nasal microbiome increased (12.4 ± 1.0 to 16.9 ± 5.6 pd in the gentamicin group; 10.2 ± 1.4 to 11.8 ± 3.1 pd in the ciprofloxacin group). No relevant difference in the phylogenetic diversity index could be observed in the two povidone groups. A schematic representation of these results is shown in Figure 2.

In contrast to the nasal microbiome, none of the administered eye drops had a relevant effect on the pharyngeal microbiome (data resp. figures on the pharyngeal microbiome are provided in the Supplement Figures S1–S4).

The most common species detected by MALDI-TOF were *Streptococcus* spp. and *Staphylococcus* spp., among others. In half the cases (50%), the MIC values of *Staphylococcus* spp. were ≥0.125 mg/L for gentamycin which indicate resistant strains, only certain isolates showed elevated MICs (1–2-fold) after treatment compared to baseline (~11%) within both antibiotic groups. Similar results were seen for *Streptococcus* spp. with E-tests of single and pooled isolates, as MICs of both antibiotics tended to be elevated after treatment (~18% of the isolates, microbiological). Nevertheless, these MIC elevations (1–2 fold) are within the uncertainty of MIC testing, indicating no antibiotic resistance development.

**Figure 3:**
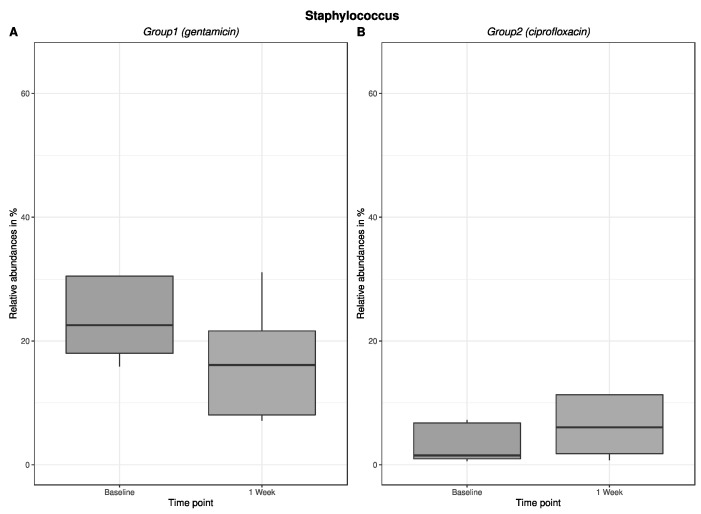
The change in relative abundance in % (*Staphylococcus*/nasal) after 1-week use of gentamicin or ciprofloxacin eye drops. Figure (**A**) shows the change in relative abundance in % in group 1, which received gentamicin for one week. Figure (**B**) shows the change in relative abundance in % in group 2, which received ciprofloxacin for one week.

**Figure 4:**
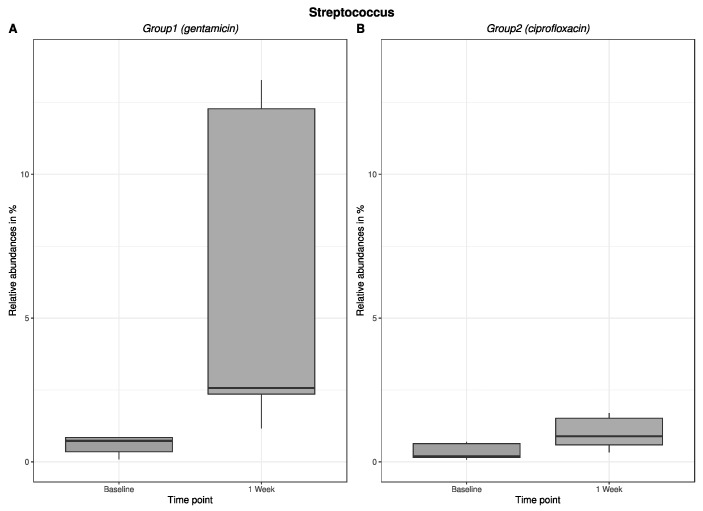
The change in relative abundance in % (*Streptococcus*/nasal) after 1-week use of gentamicin or ciprofloxacin eye drops. Figure (**A**) shows the change in relative abundance in % in group 1, which received gentamicin for one week. Figure (**B**) shows the change in relative abundance in % in group 2, which received ciprofloxacin for one week.

An excerpt of our results regarding the change in relative abundance of the major bacterial genera in the antibiotic groups in the nasal bacterium is shown in Figure 3: *Staphylococcus*, and Figure 4: *Streptococcus*. Here, the change in relative abundance is germ-specific—for example, decreasing for staphylococci after gentamicin treatment and increasing for ciprofloxacin. For streptococci, the results showed an increase in relative abundance after antibiotic administration. Figures about the change in relative abundance (in %) of *Staphylococcus* (Figure 3) and *Streptococcus* (Figure 4) in the pharyngeal bacterium are provided in the supplement.

## 4. Discussion

In healthy subjects, we discovered that use of antibiotic eye drops for one week had an influence on the nasal microbiome. This is of relevance due to the fact that various illnesses, especially respiratory diseases, including allergic or chronic rhinosinusitis with nasal polyps, asthma, bronchitis, or influenza have been linked to changes in the nasal microbiome [29]. Studies detected correlations between the alteration of the nasal microbiome and the occurrence of respiratory diseases, hypothesizing that a dysbiosis of the nasal microbiome may promote the onset of these diseases. Even the pathogenesis of neovascular AMD has been linked to nasal microbiome alterations. An explanation for these findings could be the anatomical proximity of the mentioned organs, which allows proinflammatory pathogens to easily spread among them and trigger chronic inflammatory diseases [11,12,13].

After antibiotic therapy, usually a numerical decrease in bacterial counts would be expected. Remarkably, the opposite was observed in the current study. A possible explanation could be that the concentration of antibiotics reaching the nose was too low. Even though studies have shown that subtherapeutic concentration or low antibiotic levels might favor development of resistance [30], this was not the case within our study. It is known that orally administered low-dose antibiotics impair the gut microbiome, which promotes the growth of pathogenic strains [31].

Antibiotic resistance was evaluated only by repeated antibiotic susceptibility testing which relies on a phenotypically evaluation. Thus, we would like to add here that antibiotic eyedrops did not lead to an increase in phenotypic resistance development (in the obtained bacterial isolates in the nose or pharynx when administered daily for 7 ± 1 days) but would like to emphasize that we did not check antibiotic resistance on molecular basis. Therefore, to evaluate if antibiotic resistance has occurred in form of resistance genes analysis on molecular basis has to be done.

In contrast to the nasal microbiome, the nearly non-existent effect of antibiotic eye drops on the pharyngeal microbiome can be caused by the greater anatomical distance. Probably the eye drops no longer or hardly reach the pharynx.

Previous studies showed that BAC has an antibacterial effect on the nasal microbiome [32]. We, therefore, expected a similar effect in this study and included two control groups that either received eye drops with or without BAC. Contrary to previous data, we observed no difference in bacterial counts in these two groups.

Since both antibiotic eye drops contained BAC as a preservative and we selected one control with BAC and one without, we can assume that this effect probably originated from the antibiotic.

Other studies have shown that antibiotic eye drops (fluoroquinolones) have an effect on the distribution of bacteria in the microbiome of the nose and conjunctiva [33]. In addition, the occurrence of resistance to the applied antibiotics’ used has already been addressed in some studies [34,35]. These studies also used a culture-based method to analyze the microorganisms. Nevertheless, with these analyzing methods, it is possible to detect only a few specific species of bacteria, highlighting the importance of the subsequent microbiome analysis in our study.

In the present study, a relatively new sequencing method, “next generation sequencing” (NGS) was used, which can obtain more accurate information about the bacteria, the composition, and structure of the nasal/pharyngeal microbiome by identifying the entire bacterial microbiome at once. In culture-based systems, the intended species has to be determined in advance. NGS, therefore, saves several analysis steps, while providing all relevant information about the present microorganisms [24,33,36,37,38,39].

Several other groups used the advantage of NGS technology to analyze microbiota, e.g., to analyze changes of the ocular surface microbiome after contact lens wear, diabetes, or dry eye [39]. Furthermore, NGS was also used to investigate changes in the oral microbiome after periodontal interventions [40].

However, it has to be kept in mind that the present study only included five subjects per group and only had a pilot character. Moreover, it must be said (despite very similar microbiota) that interindividual variations of microbiota are not decisive here; rather, the total value of change has significance. Another limitation that should be taken into consideration is the fact that no measurements of the concentration of the antimicrobial agent have been performed at the target sites, due to technical feasibility.

Another important point is the aspect of pneumococcal vaccination and the influence on possible change in the composition of the nasopharyngeal microbiome. These vaccines, but also other external interventions, such as the use of antibiotics, can lead to dysbiosis of the nasopharyngeal microbiome and are associated with a disbalance in the ratio between commensal and potentially pathogenic bacteria. The impact of vaccination on the microbiome is still poorly understood [41,42].

According to a study examining the effect of 10-valent pneumococcal conjugate (PCV10) vaccination on the nasopharyngeal microbiome, analysis found that bacterial composition was similar between the unvaccinated and vaccinated subjects. In addition, bacterial diversity did not differ between the vaccinated and non-vaccinated subjects. The study showed that the PCV10 vaccine works without significantly altering the nasopharyngeal microbiome. However, a higher abundance was observed in patients after PCV-10 vaccination. The genera *Streptococcus* and *Haemophilus* were increased in the vaccinated group while *Moraxella* was decreased (not statistically significant) [42].

In principle, we can primarily disregard this aspect tangentially since we assume the prevailing microbiome of the subjects as the initial value and the potential change was of interest. The primary composition of the microbiome was not the main focus of this study; thus, theoretically, a possible childhood vaccination can be hypothetically ignored, since we explicitly looked at the change from baseline to the state after a one-week antibiotic eye drops instillation period.

In order to confirm the present results, future studies with a larger sample size and a longer follow-up period are needed. These studies may also answer whether the observed changes in the microbiome are transient, and if yes, how long they last. For this purpose, this pilot study with 20 subjects was carried out, which is described in the manuscript. The control and the follow-up period for the subsequent main part of the study will be selected on the basis of the results collected. These results also provide data to generate calculations regarding the sample size of the upcoming study.

Based on the results, we have discovered that antibiotic eye-drops might have an effect on the nasal microbiome. This is the first study demonstrating that topical treatment with an antibiotic agent can unintentionally impact bacterial microbiome in anatomical proximity places. In contrast, none of the administered eye drops had a relevant effect on the pharyngeal microbiome.

To conclude, antibiotic eye drops led to an increase in the diversity and richness of bacterial strains in the nose when administered daily for 7 ± 1 days. In order to confirm these results, larger studies with a longer observation period are needed.

## Figures and Tables

**Table 1:** Description of the four study groups (*n* = 5 subjects/group).

Group	Study Medication	Age (Years ± SD)	Sex (m/f)
1	Gentamicin eye drops	26 ± 9	1/4
2	Ciprofloxacin eye drops	29 ± 4	1/4
3	Povidone eye drops unpreserved	29 ± 5	1/4
4	Povidone eye drops preserved with BAC	24 ± 4	1/4

## Data Availability

Paired-end FASTQ raw sequence reads were uploaded to the NCBI sequence read archive (SRA) and can be accessed under the BioProject ID PRJNA851930.5.

## Supplementary Materials

The following supporting information can be downloaded at: https://www.mdpi.com/article/10.3390/antibiotics12030517/s1, Figure S1: Microbial richness in pharyngeal samples; Figure S2: Faith_pd in pharyngeal samples, meaning the sum of the branch lengths of a phylogenetic tree connecting all species in the target assemblage; Figure S3: The change in relative abundance in % (*Staphylococcus*/pharyngeal) after 1-week use of gentamicin or ciprofloxacin eye drops; Figure S4: The change in relative abundance in % (*Streptococcus*/pharyngeal) after 1-week use of gentamicin or ciprofloxacin eye drops.
