# Supplementary material for: Effect of Antibiotic Eye Drops on the Nasal Microbiome in Healthy Subjects—A Pilot Study
Source: Antibiotics (Basel). 2023 Mar 4;12(3):517. doi: 10.3390/antibiotics12030517 (PMC10044076; doi:10.3390/antibiotics12030517)
Supplement: Supplementary file 1 [file antibiotics-12-00517-s001.zip › antibiotics-2179072-supplementary.pdf]

Effect of Antibiotic Eye Drops on the Nasal Microbiome in Healthy Subjects – A pilot study

Supplement

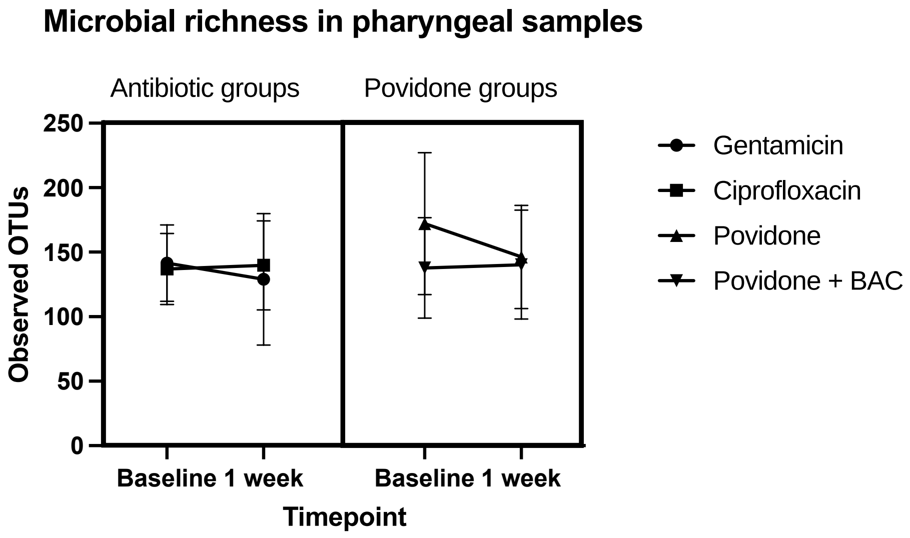

Figure S1. Microbial richness in pharyngeal samples. Comparison of OTUs before and after treatment (Baseline: before instillation of eye drops, 1 week: after 7 days instillation). Sequences are clustered according to their similarity to another. Group 1 received gentamicin eye drops, group 2 ciprofloxacin, group 3 topical lubricants containing povidone only, group 4 received topical lubricants containing povidone and BAC.

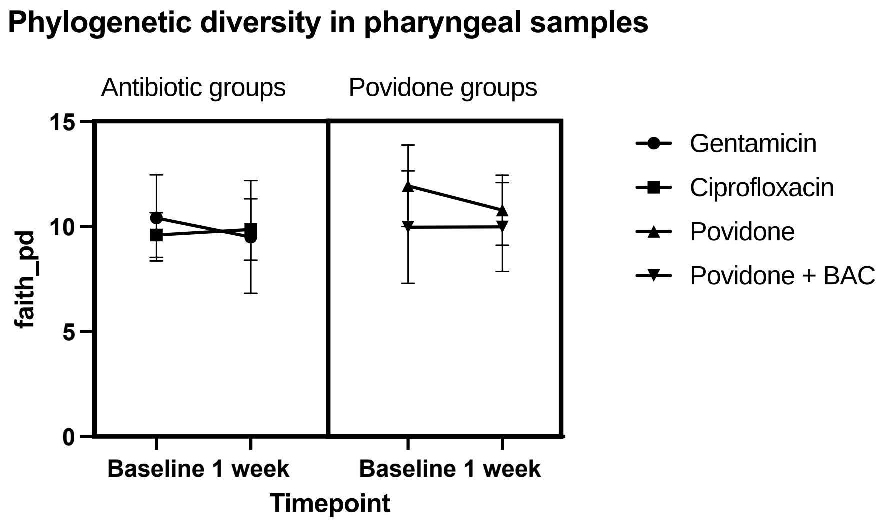

Figure S2. Faith_pd in pharyngeal samples, meaning the sum of the branch lengths of a phylogenetic tree connecting all species in the target assemblage. Comparison of the phylogenetic diversity index before and after treatment (Baseline: before instillation of eye drops, 1 week: after 7 days instillation). Group 1 received gentamicin eye drops, group 2 ciprofloxacin, group 3 topical lubricants containing povidone only, group 4 received topical lubricants containing povidone and BAC.

Effect of Antibiotic Eye Drops on the Nasal Microbiome in Healthy Subjects – A pilot study

Supplement

An excerpt of our results regarding the change in relative abundance of the major bacterial genera in the antibiotic groups in the **p**haryngeal bacterium is shown in figure 3: Staphylococcus, and figure 4: Streptococcus.

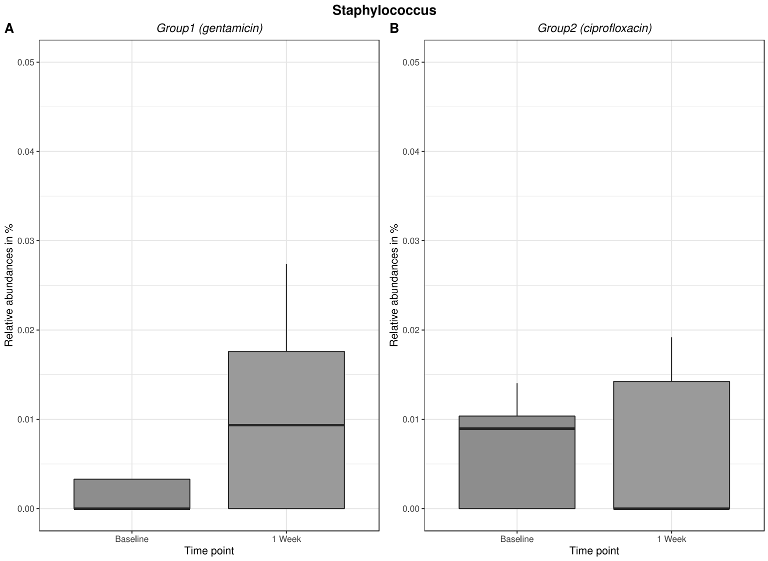

Figure S3: The change in relative abundance in % (Staphylococcus/pharyngeal) after 1-week use of gentamicin or ciprofloxacin eye drops. Figure **A** shows the change in relative abundance in % in group 1, which received gentamicin for one week. Figure **B** shows the change in relative abundance in % in group 2, which received ciprofloxacin for one week.

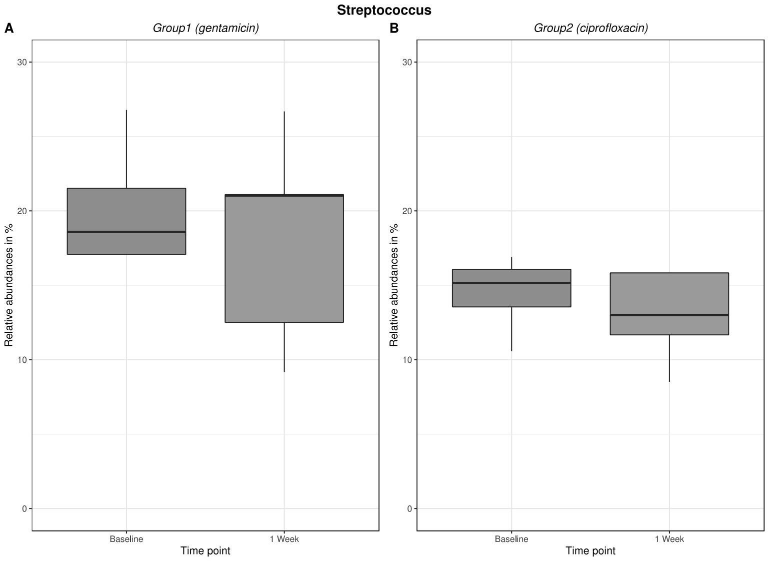

Figure S4: The change in relative abundance in % (Streptococcus/pharyngeal) after 1-week use of gentamicin or ciprofloxacin eye drops. Figure **A** shows the change in relative abundance in % in group 1, which received gentamicin for one week. Figure **B** shows the change in relative abundance in % in group 2, which received ciprofloxacin for one week.
